# Faecal short-chain fatty acid pattern in childhood coeliac disease is normalised after more than one year's gluten-free diet

**DOI:** 10.3402/mehd.v24i0.20905

**Published:** 2013-09-25

**Authors:** Bo Tjellström, Lotta Högberg, Lars Stenhammar, Karin Fälth-Magnusson, Karl-Erik Magnusson, Elisabeth Norin, Tommy Sundqvist, Tore Midtvedt

**Affiliations:** 1 Department of Microbiology, Tumor and Cell Biology, Karolinska Institute, Stockholm, Sweden; 2 Department of Paediatrics in Norrköping, County Council of Östergötland, Norrköping, Sweden; 3 Division of Paediatrics, Department of Clinical and Experimental Medicine, Faculty of Health Sciences, Linköping University, Linköping, Sweden; 4 Department of Paediatrics in Linköping, County Council of Östergötland, Norrköping, Sweden; 5 Division of Medical Microbiology, Department of Clinical and Experimental Medicine, Faculty of Health Sciences, Linköping University, Linköping, Sweden

**Keywords:** children, coeliac disease, gluten free diet, faecal short chain fatty acids, gut microflora

## Abstract

**Objective:**

Recent work indicates that the gut microflora is altered in patients with coeliac disease (CD). Faecal short-chain fatty acids (SCFAs) are produced by the gut microflora. We have previously reported a high SCFA output in children with symptomatic and asymptomatic CD at presentation, as well as in CD children on a gluten-free diet (GFD) for less than 1 year, indicating deviant gut microfloral function. In this report, we focus on faecal SCFA production in coeliacs on GFD for more than 1 year.

**Materials and methods:**

Faecal samples were collected from 53 children with CD at presentation, 74 coeliac children on GFD for less than 1 year, and 25 individuals diagnosed with CD in childhood and on GFD for more than 1 year. The control group comprised 54 healthy children (HC). The faecal samples were analysed to show the SCFA pattern taken as a marker of gut microflora function. We applied a new fermentation index, reflecting the inflammatory activity of the SCFAs (amount of acetic acid minus propionic acid and *n*-butyric acid, together divided by the total amount of SCFAs).

**Results:**

In coeliacs on GFD for more than 1 year, the individual SCFAs, total SCFA, and fermentation index did not differ significantly from the findings in controls. In contrast, the faecal SCFA level was clearly higher in coeliacs treated with GFD for less than 1 year compared to those more than 1 year.

**Conclusions:**

This is the first study on SCFA patterns in faecal samples from individuals with CD on GFD for more than 1 year. Our study indicates that the disturbed gut microflora function in children with CD at presentation and after less than 1 year of GFD, previously demonstrated by us, is normalised on GFD for more than 1 year.

Coeliac disease (CD) is characterised by small-bowel mucosal inflammation caused by wheat gluten or related prolamines in rye and barley, affecting genetically predisposed individuals carrying the HLA-DQ2 or -DQ8 haplotypes (1, 2). Treatment with a gluten-free diet (GFD) leads to normalisation of the enteropathy. Recent work indicates that the gut microflora plays an important role in the pathogenesis of CD (3–6). We proposed further evidence of intestinal dysbiosis after analysing faecal short-chain fatty acids (SCFA) that are produced by the gut flora. We reported that the SCFA patterns in children with untreated CD and CD children on GFD for up to 1 year, differ from healthy controls, reflecting the perturbed gut microflora in CD (7, 8).

In this report, we focus on faecal SCFA production in coeliacs on GFD for more than 1 year, comparing this with children with newly diagnosed CD, coeliac children on GFD for less than 1 year and healthy controls, respectively.

## Materials and methods

### Patients and controls

The clinical part of the study was performed at the Paediatric Clinic, Norrköping Hospital, Norrköping, Sweden, between 1998 and 2006. The study group comprised children consecutively diagnosed with CD, based on small-bowel biopsy findings according to criteria formulated by the European Society for Paediatric Gastroenterology, Hepatology and Nutrition (9), including information regarding serum antibodies towards gliadin, endomysium, and/or tissue transglutaminase.

Data on the patients studied are presented in Table 1. The following groups were studied: Group A, 53 children with CD at presentation, that is on a normal gluten-containing diet, with positive coeliac serology markers and small-bowel biopsy showing enteropathy compatible with CD; Group B, 74 coeliac children on GFD for less than 1 year; Group C, 25 individuals diagnosed with CD in childhood and on GFD for more than 1 year (median: 4 years; range: 13 months to 19 years). For comparison, we used results from 54 healthy children (HC) (Group D). The HC children were recruited from child welfare clinics or schools in the town of Norrköping. They were all on a normal, gluten-containing diet and showed no signs of malnutrition.

**Table 1:** Data on patients studied and results of gut microbiota activity, as described by short chain fatty acid (SCFA) concentrations and SCFA fermentation index in individuals with coeliac disease and healthy controls

Group	A CD at presentation	B CD on GFD<1 year	C CD on GFD >1 year	D Healthy controls
No. of individuals	53	74	25	54
Age (years) (median; range)	6.7 0.5–17.5	4.5 1.0–17.5	21.5 2.5–32.5	3.5 0.25–15.5
Male/female	20/33	27/47	9/16	28/26
Number of of faecal samples	53	107	28	126
SCFA§				
Acetic acid	71.6±7.4**	69.1±3.7**	30.9±3.6	27.8±1.1
Propionic acid	15.7±1.3	15.2±0.8**	12.2±0.9	11.7±0.5
*i*-Butyric acid	2.5±0.2**	2.4±0.1**	2.4±0.3	1.7±0.1
*n*-Butyric acid	19.3±2.0	16.2±0.9	13.3±1.8	15.0±1.0
*i*-Valeric acid	3.3±0.2**	3.1±0.2**	2.9±0.4	2.1±0.1
*n*-Valeric acid	1.8±0.2	2.0±0.1**	1.8±0.2	1.4±0.1
Total SCFAs	114.7±10.2**	108.5±4.9*	64.0±4.1	60.2±2.1
Fermentation index¤	0.29±0.03**	0.33±0.02**	0.07±0.06	0.05±0.02

CD, coeliac disease; GFD, gluten-free diet; SCFA, short-chain fatty acid.

§ Mean (mmol/kg faeces)±SEM.

*^/^**Significant difference vs. controls

* *p*<0.05

** *p*<0.01.

¤ Fermentation index is the amount of acetic acid minus propionic acid and *n*-butyric acid, together divided by the total amount of SCFAs.

None of the individuals in this study had been treated with antibiotics within 3 months prior to the faecal sampling. CD children at presentation delivered faecal samples before they started a GFD. In the coeliacs on GFD and the HC, faecal samples were taken on 1–4 occasions with 3-month intervals over a period of 1 year without any dietary changes regarding gluten intake.

### Microflora-associated characteristics

A microflora-associated characteristic (MAC) is defined as the recording of any anatomical structure, physiological, biochemical, or immunological function in an organism that has been influenced by the microflora (10). In this study, we used the faecal SCFA pattern as MAC.

The faecal samples were frozen either immediately or at least within 20 min of passage and stored at −20°C for pending analysis. SCFA analyses were performed at the Karolinska Institute, Stockholm, Sweden. The faecal material was homogenised after addition of distilled water containing 3 mmol/L of 2-ethylbutyric acid (internal standard) and H_2_SO_4_ (0.5 mmol/L). A 2-mL sample of the homogenate was vacuum distilled according to the method of Zijlstra et al. (11), modified by Höverstad and Björneklett (12). The distillate was analysed using gas–liquid chromatography and quantified using internal standardisation. Flame ionisation detection was employed. The results were expressed in mmol/kg wet weight. The following SCFAs were analysed: acetic acid, propionic acid, *i*-butyric acid, *n*-butyric acid, *i*-valeric acid, and *n*-valeric acid.

### Statistical analysis

Statistical analysis was performed using Student's *t*-test with Bonferroni correction. *p*<0.05 (*) and *p*<0.01 (**) were considered significant.

### Ethical considerations

The Research Ethics Committee of Linköping University approved this study.

## Results

The results are presented as: (1) individual and total faecal SCFA concentration; and (2) a fermentation index (amount of acetic acid minus propionic acid and *n*-butyric acid, together divided by the total amount of SCFAs). The fermentation index reflects gut bacterial fermentation mainly of carbohydrates. A low index indicates high anti-inflammatory activity.

**Fig. 1:**
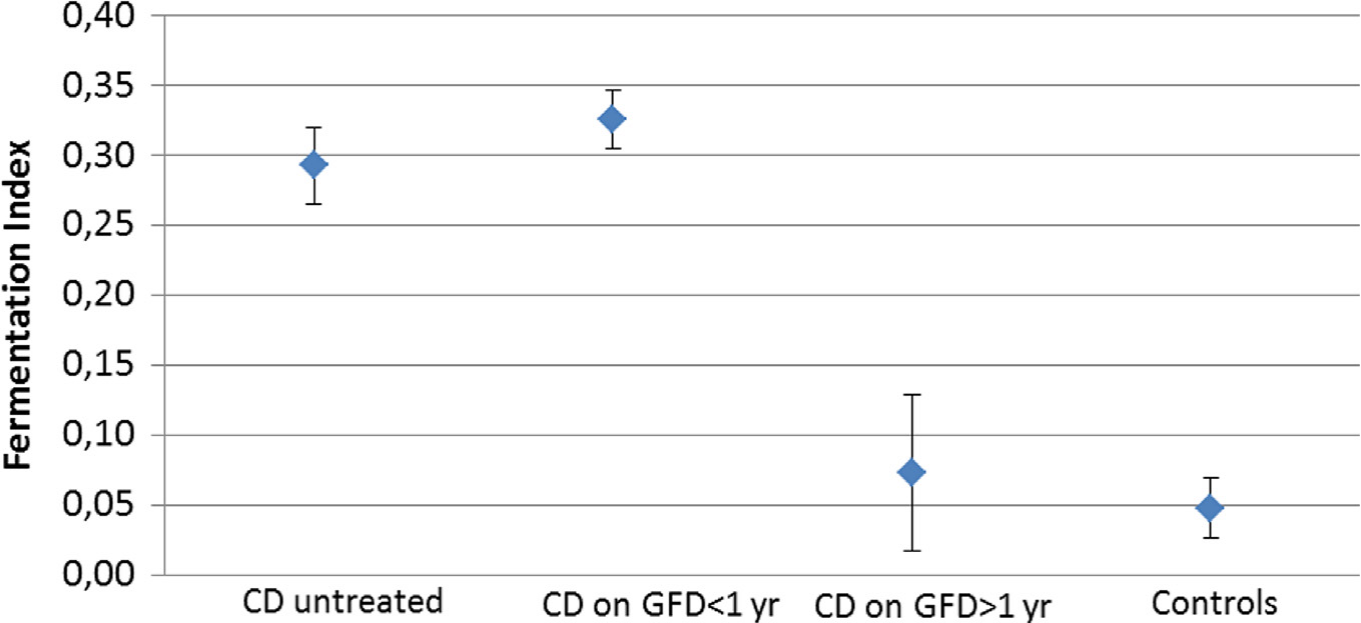
Display of short-chain fatty acid (SCFA) fermentation index (see Table 1) in individuals with coeliac disease (CD) at presentation, after less than 1 year of gluten-free diet (GFD), more than 1 year of GFD and healthy controls, respectively. The values are expressed as mean±SEM.

CD children at presentation and coeliac children on GFD for less than 1 year had significantly higher acetic acid, *i*-butyric acid, *i*-valeric acid, total SCFA, and fermentation index than healthy controls (Table 1; Fig. 1) (*p*<0.01). In coeliacs on GFD for more than 1 year, the individual SCFAs, total SCFA, and fermentation index did not differ significantly from controls. There was a clear difference between coeliacs treated with GFD for less than 1 year, and those who had been on the GFD for more than 1 year regarding acetic acid, total SCFA, and fermentation index.

## Discussion

This is the first report on the faecal SCFA profile in patients with childhood CD treated with GFD for more than 1 year. The study shows that this group of coeliacs has the same low total SCFA concentration as healthy controls. This finding of a normalisation following more than 1 year on a GFD is very much in contrast to our previous reports of high faecal SCFA output in children with symptomatic (7) and asymptomatic (8) CD at presentation as well as in CD children on GFD for a short period of time (7). As SCFAs are produced by the gut microflora, our previous and present findings indicate that children with untreated CD and coeliac children with a brief GFD treatment have a deviant gut microflora, which returns to normal after more than 1 year on GFD. This is in agreement with a recently published report by Bertini et al. (13) analysing serum and urine. They found a characteristic, partly gut flora-derived, metabolic signature in patients with untreated CD, which returned to normal in CD patients treated with GFD for 12 months. Taken together, our findings and those by Bertini et al. suggest that the disturbed gut microflora function in untreated CD is not a permanent condition, but may well be normalised by a GFD of longer duration. Thus, the 1-year period on a GFD, commonly accepted as the normal period required for children with newly diagnosed CD to recover, does not seem to be long enough to normalise the gut flora functions.

Interestingly, Caminero et al. (14) recently reported on the activity of intestinal microbiota by analysing the faecal SCFA pattern in 11 healthy young adults on normal gluten-containing diet, after a period of GFD and GFD plus various amounts of gluten, respectively. They found significant changes in the SCFA pattern in subjects on an unphysiologically high gluten intake only. Thus, the faecal SCFA profile did not differ between subjects on normal diet and GFD. Consistent with this, we found similar SCFA concentrations in healthy controls and CD patients on GFD >1 year but not in those on a shorter GFD. It seems, therefore, that normalisation of the gut microflora metabolic activity in CD patients on GFD is a time-dependent process.

The appearance of SCFA in faeces reflects a complex interplay between host, diet and microbes. Changes in SCFA levels may be the result of altered gut microflora. However, faecal SCFA concentrations also depend on the availability of fermentable substrates. Untreated CD is characterised by villous atrophy and a reduced capacity to absorb nutrients, which leads to increased delivery of fermentable substrate to the colon. This may explain higher levels of SCFA in faeces in coeliacs treated with GFD for less than 1 year and accordingly reduced levels in patients treated for more than 1 year, reflecting healing of the small intestinal mucosa.

Acetic acid, *n*-butyric acid, and propionic acid are the three most common SCFAs derived from bacterial fermentation of dietary carbohydrates. It is well established that both butyric acid in particular, and propionic acid, have positive metabolic effects on enterocytes, while acetic acid is pro-inflammatory (15–17). In addition, butyric acid has proved effective in treating gastrointestinal mucosal inflammation (18). In order to briefly illustrate the inflammatory properties of faecal SCFAs in children with Crohn′s disease, we recently reported the use of a fermentation index (amount of acetic acid minus propionic acid and *n*-butyric acid, together divided by the total amount of SCFAs) (19). As both Crohn's disease and CD are prototypic disorders of chronic gastrointestinal mucosal inflammation as a response to changes in the gut microflora (20), we used the same index to elucidate gut-microbiota-mediated alterations in CD children in different phases of the disease. In the coeliac individuals treated with GFD for more than 1 year, the low fermentation index did not differ from the controls, primarily due to the decreased production of acetic acid. Taken together, these findings suggest that this simple faecal SCFA index may be useful in illustrating the activity of chronic gut microflora-derived mucosal inflammation.

It can be speculated that the altered microbial fermentation, as illustrated in this study, could be involved in the generation of intestinal symptoms in CD, in accordance with the findings in patients with irritable bowel syndrome (21). Even extra-intestinal symptoms due to SCFA fermentation products of the microbiome have been implicated in autism spectrum disorders (22).

The coeliacs treated with GFD for more than 1 year (Group C) were older than the other coeliacs and healthy controls studied. However, all patients in Group C had a childhood CD diagnosis. Thus, we consider the study groups comparable, since it has been shown that children have established a permanent gut microflora by 2 years-of-age (23). Further evidence is produced by the mere fact that the SCFA results in Groups C and D are very similar to those in adults (23).

Kalliomäki et al. (24) recently reported altered gene expression of toll-like receptors in the small intestinal mucosa of children with CD, suggesting that microbiota-associated factors might potentiate the effects of gluten proteins in the molecular pathogenesis of the disease. Most previous work on the intestinal microflora in patients with CD has focused on identifying specific bacteria in intestinal mucosal biopsies or faecal samples from patients with CD (5). Undoubtedly, it is of interest to characterise deviations in the gut microflora in coeliac patients compared to controls in order to elucidate its eventual role in the pathogenesis of the chronic intestinal mucosal inflammation characteristic of CD (3, 4). Regrettably, findings in humans have so far been either inconsistent or difficult to interpret. Interestingly, oral administration of *Lactococcus lactis*, capable of secreting DQ8-restricted gliadin epitope, has been shown to significantly inhibit systemic immune responses in a mouse model (25). If this also holds true for humans, such microbe-based manipulation might be a future option in the treatment of CD (26).

## Conclusion

In summary, our study indicates that the deviant gut microflora function, demonstrated in children with CD at presentation and after a short period of gluten-free treatment, is normalised on GFD but only after more than 1 year. To our knowledge, this is a new observation not previously reported.
